# The applicability of the European GMO legislation to epigenetically modified organisms

**DOI:** 10.3389/fbioe.2023.1124131

**Published:** 2023-02-27

**Authors:** Timo Faltus

**Affiliations:** Faculty of Law, Economics and Business, Martin-Luther-University Halle-Wittenberg, Halle an der Saale, Germany

**Keywords:** epigenetically modified organisms, epigenetic techniques, crops, European GMO legislation, directive 2001/18/EC, legal classification

## Abstract

In addition to classic genetic engineering for the targeted modification of the base sequence of the DNA, epigenetic methods for the targeted modification of the genetic material without base changes are increasingly being used. Such epigenetic techniques can be used, for example, to influence stress tolerance to heat or aridity in plants. The regulatory handling of organisms generated by means of epigenetic techniques on the grounds of genetic engineering law has not yet been clarified. This paper critically reviews the legal classification of epigenetically modified organisms as GMOs as expressed in the study on New Genomic Techniques published in April 2021 by the European Commission. The paper shows that there are reasons to assume that epigenetically modified organisms are not covered by the European GMO legislation. In addition, the paper provides an introductory overview of the significance of epigenetics and the methods used to intentionally influence epigenetic traits and illustrates the possibility for a consistent, risk-based regulation of epigenetic modifications.

## 1 Introduction

In April 2021, the European Commission published a study regarding the status of New Genomic Techniques (NGTs) under Union law (European Commission, 2021)—below referred to as “the study.” The Council of the European Union had asked for this study regarding the status of NGTs under Directive 2001/18/EC, Regulation (EC) 1829/2003, Directive 2009/41/EC and Regulation (EC) 1830/2003 and in light of the European Court of Justice’s judgment in Case C-528/16 (Court of Justice of the European Union, 2018).

NGT is not a legally fixed term and is used as a generic term for techniques that allow for targeted modifications of the genetic makeup of various organisms. In the scientific, technical understanding the term NGT typically includes methods such as site-directed nuclease technology (SDN), oligonucleotide-directed mutagenesis (ODM), cisgenesis, and intragenesis. Some of the legal discourse on the regulatory handling of these methods focuses not only on the methodological procedure for classification as NGT, but also on a temporal aspect based on Directive 2001/18/EC. According to this, only such techniques for the targeted modification of genetic traits are to be classified as NGTs if they were developed after the year 2001. The methods available before are therefore classic or established (old) genomic techniques.

So far, the discourse on the legal classification of organisms as GMOs following the application of NGTs has focused on those techniques that influence the base sequence of the DNA. Now, however, there are also techniques such as RNA-dependent DNA methylation (RdDM) available for the targeted manipulation of epigenetic properties, so that legal questions also arise about the regulatory handling of epigenetically modified organisms.

With regard to the wording of Directive 2001/18/EC the European Commission’s study recalls that an organism obtained through NGTs is an organism whose genome has been altered in a way that does not occur naturally by mating and/or natural recombination, thus a GMO. Therefore, such a GMO is subject to the provisions of the GMO legislation. In addition, the study recommends that even organisms in which only epigenetic modifications have been introduced without also requiring a change in the base sequence of the DNA should legally be qualified as GMOs. At the same time, the study emphasizes that NGTs can advance the sustainability of agricultural production in line with the goals of the green deal and farm-to-fork strategy.

In view of the growing practical significance of epigenetic techniques in agriculture, this paper aims to deliver a background on epigenetic techniques focusing on DNA modifications by means of DNA methylation and histone modifications and their use in plant breeding. It is shown that, on the one hand, there are genetic engineering techniques that can achieve the epigenetic change only together with a change in the base sequence of the DNA, and that, on the other hand, there are techniques that can change the epigenetic constitution without also requiring a change in the base sequence of the DNA. Based on these technical descriptions, the paper aims to critically review the legal interpretation in the study based on the biological facts and various provisions of the GMO legislation.

## 2 Epigenome and epigenetics

### 2.1 Epigenetic terms

There is no legally agreed definition of what the epigenome may comprise or what techniques are described by the term epigenetic(s). Within the scientific discourse the term epigenome typically describes the entirety of chemical modifications of the DNA and histone proteins of an organism that do not affect the base sequence of the DNA. One often-cited wording coins epigenetic modifications as “*the structural adaptation of chromosomal regions so as to register, signal or perpetuate altered activity states*” (Bird, 2007) or “*mitotically and/or meiotically heritable changes in gene function that cannot be explained by changes in DNA sequence*” (Riggs et al., 1996; Riggs and Porter, 1996).

According to the high level group of scientific advisors to the Scientific Advise Mechanism of the European Commission (SAM), epigenetics “*describes information encoded in chromosomes, but not directly in the DNA sequence, which contributes to the determination of stable, heritable phenotype, along with the genotype and environmental factors*” (European Commission, and Directorate-General for Research and Innovation, 2017).

Epigenetic patterns in an organism can change spontaneously in response to environmental impacts. These epigenetic changes, sometimes called “epimutations,” do not change the sequence of the nucleotides of a DNA (primary structure of DNA) but lead to changes of three-dimensional folding characteristics of that DNA and its interaction with associated proteins (quaternary structure), that may alter the transcriptional expression of genes at least within the next-generation. These epigenetic properties or changes can then also be reflected in (different) phenotypic properties (cf. 3.1).

The New Techniques Working Group of the Competent Authorities stressed in its final document to the European Commission the fact that a stable phenotype caused by epigenetic changes “*may remain in the progeny for some generations only …* ” and “*in some applications (it) may be that the epigenetic changes fade out and, in that sense, the duration of the effect is unpredictable over time*” (New Techniques Working Group, 2012).

### 2.2 DNA-methylation

DNA can be methylated by binding a methyl group (-CH_3_) to cytosine (C). Methylation and demethylation are the natural cause of some phenomena of non-mendelian inheritance (Li et al., 2013).

### 2.3 Histone modification

Histones serve as spools around which DNA can wind to put the DNA string into a more condensed structure. The degree of DNA condensation, i.e., the degree of how intensively the DNA molecule is folded and wrapped up with the help of histones, determines its accessibility and biological activity.

This entity of folded DNA and protein, called chromatin, may undergo a structural change by introducing chemical modifications, or removing them, and, hence, genes embedded in these regions may open up and be exposed to the transcriptional machinery. There is a natural, dynamic transition between active and repressed chromatin structure/states that regulates gene expression underlying developmental processes and responses during stress conditions (Kim et al., 2015).

Histones exist in many genetic variants. The N-terminal and C-terminal tails of histone proteins can be chemically modified by enzymes among which methylation, acetylation and phosphorylation are the most relevant ones. These histone modifications can be reversed by specific enzymes, enabling dynamic changes in chromatin states. Most studies focus on the methylation and acetylation state.

### 2.4 Unique aspects of epigenetic modifications

Similar to genetic mutations, epigenetic modifications can result in heritable changes in gene expression and function. However, there are several characteristics unique to epigenetic modifications that differ from traditional genetic mutations in which the DNA sequence is changed.

Most important is the reversible nature of epigenetic modifications. All DNA- and histone modifications are intrinsically reversible (Herceg and Hainaut, 2007; Pikaard and Mittelsten Scheid, 2014). In contrast, genetic mutations tend to be irreversible, i.e., that once mutations have occurred, they typically do not mutate back again (cf. Kovalchuk et al., 2000).

Another unique feature of epigenetic changes is the tendency to be tissue-specific (Widman et al., 2014) whereas genetic changes tend to be systematic. The epigenetic pattern within a single organism can differ from 1 cell type to another. In distinction to this, germline inherited genetic mutations effect the whole organism, whereas spontaneous somatic mutations effect only individual cells.

However, the genetic information itself, i.e., the particular nucleic acid sequence leading to certain protein in the case of transcription/translation, is untouched by epigenetic modifications and can be inherited unchanged. Moreover, it is a qualitative difference whether altered genetic information itself is inherited or whether only factors are inherited, influencing the “use” of the unchanged genetic information. Likewise, such factors exist as proteins in the egg cell’s cytoplasm, which are taken over by the zygote and select the genes being transcribed in the early development of the new organism. In other words, it is important to distinguish heritable changes that arise from sequence changes in DNA from those that do not.

## 3 Application and detection

### 3.1 Practical meaning of epigenetic modification techniques for agriculture

To tackle fluctuating growth conditions and environmental stresses, plants in various ways use epigenetic modifications to acquire quick adaptation/response and phenotypic/developmental plasticity. Therefore, the modification of epigenetic factors harbors the potential for crop improvement, namely, epi-breeding (Kakoulidou et al., 2021).

In several crops, traits have been identified caused by natural epigenetic variability, e.g., dwarf phenotypes in rice, an increased seed protein content and decreased oil content in oilseed rape or fruit ripening in tomato (summarized in Latutrie et al. (2019)). Such variability can be triggered by applying chemicals or using environmental stressors, or a combination of both.

The role of histone modifications has been assessed in plant response to environmental stresses, such as drought, salt and disease resistance (Roca Paixão et al., 2019; Yung et al., 2021; Zhi and Chang, 2021). In the case of drought tolerance, the chromatin embedding a drought specific regulator gene was loosened with the help of acetylation of the linked histones by epigenetic editing (Roca Paixão et al., 2019). However, histone modifications are, at the best of present knowledge, not stably transmitted during sexual reproduction (Zhi and Chang, 2021). Hence, any technique exploiting different histone modifications is limited to vegetatively propagated crops (e.g., potato, strawberry, banana, oil palm tree) so far.

As mentioned above, the transmission of epigenetic variability by sexual propagation to the next generations is usually unstable. This is due mainly to demethylation of the DNA during replication if the environmental stimulus for methylation disappeared. With vegetatively propagated crops, however, clonal and hence non-sexual propagation can be used to stabilize the desired traits. On the other hand, stable inheritance of hypermethylated DNA status has been observed for eight generations in *Arabidopsis* (Dalakouras and Vlachostergios, 2021). The mechanistic of determining the status of being either a transient or a rather stable epigenetic change is still a focus of research.

### 3.2 Targeted application of epigenetic techniques

Epigenetic techniques allow both analysis and targeted manipulation of the epigenetic status of the DNA. This includes DNA methylation, DNA-protein interactions, and histone and/or chromatin modifications within a cell or organism.

The boom of epigenetics arrived during the 1990s and 2000s with the development of cloning and biochemical techniques which allowed the identification and the use of specific enzymes, catalyzing or erasing epigenetic marks. In fact, the first DNA methyltransferase was purified and cloned in 1988 (Bestor, 1988), DNA-demethylating enzymes were firstly described in 2010 (Zhang et al., 2010). The first histone acetyltransferases and histone methylases were first described in the years 1996–1998 and 1991 to 2005, respectively (Peixoto et al., 2020).

In fact, most studies focus on gene silencing through DNA methylation: It can be accomplished in a plant by introducing a gene that, once transcribed, gives rise to the formation of double-stranded RNAs (dsRNA), which will be cleaved by the plant ribonuclease Dicer-like (DCL) into small interfering RNAs (siRNAs). If these siRNA molecules share homology with a promoter region, they can specifically induce DNA methylation resulting in transcriptional inactivation of the gene. This so-called RNA-directed DNA methylation allows for targeted gene silencing. RdDM was discovered in 1994 and since then used in research for epigenetic editing and breeding of plants (Wassenegger, 2000). Performed as described above, RdDM allowed epigenetic modifications mostly in conjunction with permanent transgenic alteration of the genome. Most recently, modified site-directed nucleases (mSDNs) have been used for epigenetic editing. After deactivation of the nuclease domains, to avoid DNA cutting, SDNs were modified to target methylases or demethylases to a specific gene and, thus, allow for site specific-change of methyl groups linked to DNA (Nuñez et al., 2021). This epigenetic editing technique allows for site specific-change of DNA methylation without permanent changes in DNA base sequence. Today, there are also virus-based RdDM methods (VIGS = virus induced gene silencing), which do not even temporarily change the DNA base sequence for targeted epigenetic modification (Dommes et al., 2019).

Furthermore, targeted epigenetic modification of histones can be achieved by recruiting de/methylation or de/acetylation factors using one of the mSDNs systems. For example, the fusion of an acetyltransferase to deactivated Cas9 (dCas9) enhanced drought stress tolerance in *Arabidopsis* plant (Roca Paixão et al., 2019). These achievements suggest that the use of this technique is in principle also possible in crops of agricultural relevance.

Finally, for the sake of completeness, methods should be mentioned that can change epigenetic properties intentionally but in an undirected manner. For this purpose, so-called “epigenetic drugs” (e.g., zebularine) are used, which affect the activity of methylases or demethylases naturally occurring in the organism. However, these approaches do not play a role in breeding because the epigenetic changes achieved with them cannot be stabilized (trans-generationally). In contrast to the generation of base mutations of DNA, radiation-based approaches to alter epigenetic properties also play no role.

The commercial application of epigenetic techniques for, e.g., plant breeding, enabled by rapid development of other molecular techniques, is the subsequent logical step and is expected to emerge in the next years as described below (Gallusci et al., 2017; Tirnaz and Batley, 2019; Gallego-Bartolomé, 2020; Varotto et al., 2020).

### 3.3 Techniques for detection of epigenetic modifications

Detection of the methylation status of DNA was achieved as early as in the 1970s. Initially, only the ratio of methylated to non-methylated DNA could be determined. Meanwhile, methods have been developed not only to determine the methylation ratio, but which can also be used to determine the exact methylated bases of the DNA (Wang et al., 2017; Šestáková et al., 2019).

In addition, today modifications of the histones can also be detected (Zhuang et al., 2020).

Although all these techniques became quite sophisticated, yet there is no method available to identify the origin of a DNA methylation or a histone modification. Hence, when detecting, e.g., a methylated cytosine within a DNA sequence there is no differentiation possible of to whether the methylation stems from the action of a natural enzyme (caused naturally, without human intervention) or of technical epi-editing. This is true when the transgene coding for the epigenome editing factors is absent after segregation, and the epi-mutation is inherited to the next generations as it has been shown (Papikian et al., 2019). This also applies to epigenetic modifications achieved by such methods that do not additionally rely on genetic engineering techniques to alter the base sequence of DNA.

Furthermore, detection methods for epigenetic modifications as such need to be robust enough to stand quality criteria being applied to analysis methods suitable to test large commodities. So far, none of these methods have been validated for such purposes. Therefore, the question of robust, validated detection methods, i.e., test methods for official, regulatory controls, must not be confused with the question of whether there are any test methods at all that can be used to investigate the epigenetic status of DNA. Furthermore, it remains questionable whether an identification of such modifications will be possible at all because, as shown, it is impossible to determine whether a certain epigenetic trait is of natural or artificial origin.

### 3.4 Off-targets in epigenome editing

Even when the used genetic engineering techniques are specific, there is the possibility of off-target effects. Off-target in genetic engineering generally means that in addition to the desired modification of the DNA, other sites can also be modified. Typically, these off-target sites have structural similarities to the on-target site. Thus, the goal of technical improvement of a genetic engineering technique is to reduce the off-targets relative to the on-target.

In terms of epigenetic modifications, for example, it has been shown that non-perfectly matching small RNAs can induce stable and heritable epigenetic modification through RdDM in plants (Fei et al., 2021). Thus, the off-target activity needs to be assessed by plant breeders to ensure the desired outcome.

### 3.5 (Unintended) epigenetic modifications in plant cell cultures

Not identical with the concept of off-targets are spontaneous (epi-) genetic modifications. It is known for a long time that any kind of cell or tissue culture propagation influences the epigenome profoundly. A number of publications have been published which show that during plant tissue culturing especially DNA methylation and histone methylation exhibit changes during dedifferentiation of the cells, for example, during plant regeneration (reviewed in Smulders and Klerk (2011) and more recently in Lee and Seo (2018)). These epigenetic modifications are also accompanied by phenotypic changes (at the cell culture level). So far, however, it seems that these undirected and random epigenetic modifications are not used for breeding purposes. In addition, a systematic assignment which of these epigenetic modifications causes exactly which phenotypic change is yet lacking.

The nature of cell or tissue culture induced epigenetic modifications seems undirected and unpredictable. Consequently, these cell culture induced modifications are indistinguishable from epigenetic modifications that are induced by other stress factors or by programmable nucleases. Despite this molecular background cell cultures are being used now for almost 70 years in conventional breeding and negative effects of somaclonal variation never posed a problem to plant breeders so far due to the selection process of the produced lines.

## 4 Sources for the legal assessment of epigenetically modified organisms

### 4.1 Findings by the new techniques working group (2012)

The RdDM technique (among numerous others) was previously looked at by the so-called “New Techniques Working Group” to assess its legal implications. The report of the “New Techniques Working Group” has not been officially published to date. However, the report circulated “unofficially” on the internet and numerous publications refer to the report. This report has thus become a discussion basis for the evaluation of NGTs and will therefore also be considered here in relation to epigenetic techniques. In 2012, this Working Group unanimously found that organisms resulting from treatment with an RdDM technique should be outside of the scope of the GMO legislation if they do not bear heritable changes in nucleotide sequences since such organisms are comparable to organisms obtained with natural processes (New Techniques Working Group, 2012, pp. 33–34).

### 4.2 European food safety agency (EFSA) statement (2015)

In 2015 the EFSA GMO Unit has stressed in response to a request by the European Commission that “*a definition of genetic material can be: the genetic material is the nucleic acid molecule as defined by its nucleic acid sequence which contains the genetic information.*” Furthermore, the EFSA GMO Unit “*did not consider scenarios which include non-sequence-related aspects, for example, those involving the modulation of the gene expression*” (European Food Safety Authority, 2015). The definition by EFSA therefore seems to not cover mere epigenetic modifications in organisms. Consequently, in its response to the European Commission, the EFSA GMO Unit made clear, that in line with the provided definition of genetic material “*the term* “*alteration of genetic material*” *could be restricted to situations where the nucleotide sequence is modified.*” EFSA’s GMO Unit has based this assessment on the fact that “*the nucleotide sequence is not altered by DNA methylation, and the modification is reversible*” (European Food Safety Authority, 2015). From these comments by the GMO Unit of EFSA, it can therefore be concluded that at least merely epigenetic technique, e.g., RdDM, which do not alter the nucleotide sequence cannot be considered as an alteration of the genetic material.

### 4.3 Findings by the Court of Justice of the European Union on NGTs (2018)

In 2018, the Court of Justice of the European Union (CJEU) was asked on the legal interpretation of the GMO definition regarding organisms modified by techniques of targeted (DNA) mutagenesis which make up the most of the NGTs.

The GMO definition is laid out in Article 2(2) of Directive 2001/18/EC and reads:

““*genetically modified organism (GMO)*” *means an organism, with the exception of human beings, in which the genetic material has been altered in a way that does not occur naturally by mating and/or natural recombination.*”

The Court held that this definition must be interpreted as meaning that organisms obtained using techniques/methods of mutagenesis constitute GMOs within the meaning of that provision (Court of Justice of the European Union, 2018, para. 38). The judges further decided that only organisms obtained by mutagenesis techniques that have conventionally been used in a number of applications and have a long safety record are covered by the exemption-clause of Article 3 (1), by which such organisms fall outside the scope of Directive 2001/18/EC. This means that, on the other hand, all organisms that are created by targeted mutagenesis are covered by Directive 2001/18/EC and are, therefore, subject to the GMO legislation and their placing on the market requires a complicated and costly approval (Court of Justice of the European Union, 2018, para. 54).

This judgment was controversially discussed especially among scientists (Faltus, 2018; Kahrmann and Leggewie, 2018; Purnhagen et al., 2018; Wasmer, 2019), since the judges as basis for their decision took up the claim by the French Conseil d`Etat according to which risks linked to the use of new techniques/methods of mutagenesis might prove to be similar to those which result from the production and release of a GMO through transgenesis (Court of Justice of the European Union, 2018, para. 48).

Therefore, in the CJEU’s line of reasoning, exempting GMOs created by targeted mutagenesis from the scope of the GMO legislation would, according to the CJEU, compromise the objective of protection pursued by the Directive 2001/18/EC and would fail to respect the precautionary principle, which it seeks to implement (Court of Justice of the European Union, 2018, para. 53).

This judgment is not binding to legal questions on mere epigenetic changes in organisms, since the CJEU ruled on targeted mutagenesis and not on epigenetic techniques. However, it is of crucial importance nonetheless: First, given the rather recent history of epigenetic techniques (cf. 3.2 above), it is likely that in light of the CJEU judgment, recital 17 of Directive 2001/18/EC could not serve as a reasoning to exclude epigenetically modified organisms from regulation, if one was to regard them as GMO, since it reads as follows:

“This Directive should not apply to organisms obtained through certain techniques of genetic modification which have conventionally been used in a number of applications and have a long safety record.”

Secondly, the ruling by the CJEU is widely, though not unequivocally (van der Meer et al., 2021), interpreted as an implicit decision for a process-based interpretation of the GMO definition (Faltus, 2018; Beck, 2019). This interpretation of the judgment and the corresponding interpretation of Article 2 (2) can easily lead to (prematurely) classifying organisms modified in any way by any new technique as GMO.

The CJEU in its judgment seemingly did not leave room for exceptions when it stated that organisms obtained through techniques/methods of mutagenesis constitute GMOs. If the CJEU judges really thought, mutagenesis does not always lead to GMO (like in cases where only a targeted single-point-mutation is introduced into the organism, which might occur naturally), then they likely would have explicitly followed the Opinion of Advocate General Bobek in that case (Court of Justice of the European Union, 2018, recital 66 of the opinion).

Yet, they did not. Therefore, the phrase “*in a way that does not occur naturally*” in the GMO-definition in Article 2 (2) of Directive 2001/18/EC cannot solely refer to the result of the genetic material’s alteration, as otherwise there would be the need for exceptions, since mutagenesis techniques in the end can indeed lead to naturally occurring genetic alterations. This approach without an exception therefore is the basis for the above mentioned process-based interpretation of Article 2 (2).

The process-based interpretation, however, does not remove all uncertainties by attempting to cover all genetic modifications: The terms “altered” and “genetic material” in Article 2 (2) are not legally defined. Nevertheless, it is obvious that both terms are of paramount importance when it comes to interpreting the legal consequences of mere epigenetic modifications. Therefore, the question remains, what “alteration” (of the genetic material) actually means in the context of the Directive 2001/18/EC.

### 4.4 Findings by the EU Commission’s study (2021)

First, it should be noted that the Commission’s study, referring back to the CJEU decision, argues that organisms obtained by means of NGTs that have appeared or have been mostly developed since the adoption of Directive 2001/18/EC are GMOs and therefore subject to the provisions of the Directive. Therefore, even though it is not explicitly addressed by the study, it must be assumed that in the understanding of the study, techniques using base modification of DNA to change epigenetic properties legally lead to a GMO, which in turn is then subject to the safety regulations of the Directive. Furthermore, the study has explicitly argued that even those organisms in which the genetic material has been altered without change of the nucleic acid sequence, in a way that does not occur naturally by mating and/or natural recombination should legally qualify as GMOs (European Commission, 2021, pp. 21–22). Therefore, in the context of the study, all RdDM techniques practically relevant for plant breeding that cause epigenetic modification (cf. 3.2) need to be legally regarded as always leading to a regulated GMO. In the study, it is held that there were no elements in the legislation that would support a restrictive interpretation of the term “alteration” in the sense of referring only to the alteration of the nucleic acid sequence of the genetic material. It is being recalled there that the CJEU followed a restrictive interpretation of mutagenesis in the context of the exemption-clause in Article 3 (1) of the Directive, i.e., only organisms created with mutagenesis-techniques which have conventionally been used in a number of applications and have a long safety record are outside of the scope of the Directive (European Commission, 2021, p. 21).

Therefore, in the study the opinion is expressed that the reasoning by the CJEU, especially the referral to the Directive’s objectives (protection of health and the environment) and the precautionary principle stressed in the Directive, also supported a wide interpretation of the term “alteration” in the sense that it would not only encompass the alteration of the nucleic acid sequence (European Commission, 2021, pp. 21–22). The exact wording in the study is:

*“The reasoning followed by the CJEU to justify its restrictive interpretation of mutagenesis in the context of the exemption […] supports a* ***restrictive*** *interpretation of the term “altered” in the GMO definition.”*

[Emphasis by the author] Clearly, this must have been an editorial mistake, as it is concluded in the next sentence that *“Organisms in which the genetic material has been altered without change of the nucleic acid sequence […] are GMOs subject to the provisions of the GMO legislation.”* This however is only possible with a **wide** interpretation of the term “altered”.

The legal interpretation for organisms with mere epigenetic modifications is much less unambiguous than the study seems to suggest. It, therefore, has to be questioned whether there are not convincing arguments to assume that such modifications that do not change the DNA-sequence indeed do not lead to a regulated GMO.

## 5 The need for a more differentiating look

Up to now, legal considerations on epigenetic modifications, especially with regard to crops, have only focused on epigenetic modifications in a general way, without clarifying whether epigenetic modifications (comparable to changes in the base sequence of the DNA) can constitute a GMO from a legal point of view. In addition, the legal discourse to date lacks a differentiated analysis of the various methods of epigenetic modification.

The appropriate legal assessment of epigenetic techniques, therefore, first must clarify whether epigenetic modifications are legally to be treated in the same way as modifications of the DNA base sequence (outcome-based assessment). If epigenetic modifications *per se* are not able to constitute a GMO from a legal point of view, then the legal analysis of the different epigenetic methods only has a limited meaning, because from the perspective of genetic engineering law there is no regulated organism.

Insofar as epigenetically modified organisms can be GMOs in the legal sense, furthermore, an appropriate legal assessment of epigenetic techniques should include a procedure-based assessment. Within this procedure-based assessment it should first be asked whether it is legally necessary due to the scientific circumstances to differentiate between techniques that work without additional modification of the DNA base sequence and those procedures that additionally rely on the modification of the DNA base sequence. Second it should be clarified whether it is legally necessary due to the scientific circumstances to differentiate between (de)methylation at the bases of the DNA and (de)acetylation/(de)methylation at the histone proteins.

To answer the mentioned questions, it is first necessary according to Article 2(2) of Directive 2001/18/EC to determine the legal scope of the terms “*genetic material*,” “*altered*” and “*modification*” by interpretation of the law, because the scope of these terms determines what is to be understood as a GMO within the meaning of the law. In the end, this clarifies, in terms of epigenetic changes, whether the following equation is applicable: altering genetic material ≙ altering DNA sequence ≙ altering epigenetic modifications. Based on this, it must then be clarified on grounds of Article 2 (2) (a,b) and Article 3 No. 1 of Directive 2001/18/EC with regard to the various epigenetic techniques whether the GMO in question is a regulated GMO or a GMO that is exempt from the Directive.

A restrictive interpretation of the terms “*genetic material*,” “*altered*” and “*modification*” could lead to epigenetic modifications falling outside the scope of the Directive. In the Commission’s study, however, it is assumed that there is no reference either in the Directive specifically or generally in the legislation on genetic engineering that would support a restrictive interpretation of the term “*altered*” in Article 2(2). This assumption is certainly debatable for the reasons described as follows.

## 6 Interpretation of the law

### 6.1 Scientific, technical premises

Generally, only the nucleic acid sequence is stable during the plant’s life cycle. The epigenetic status of the sequence is constantly changing during the plant’s life cycle following development or environmental stress conditions. The aim of methods for manipulating epigenetic properties is not only to specifically influence certain epigenetic properties, but also to actually stabilize these epigenetic properties.

By forming the DNA primary structure, i.e., the linear sequence of the bases, nucleobases build the essential part of the DNA sequence of an organism. Therefore, one could argue that the establishment of a covalent chemical bond on the nucleobase cytosine (or adenine) and thus its transformation to the new molecule methylcytosine (or methyladenine), DNA-methylation leads to a chemical modification of the nucleic acid molecule and thus consequently to changes in nucleotide sequence of an organism.

In contrast, at first glance, epigenetic changes in histones would seem to be different. It is true that the epigenetic changes in histones are also based on covalent bonds. However, the histone molecules are not covalently linked to the DNA. Thus—at least in the scientific sense—DNA and histones are two separate, independent molecules, even if both molecules exist as nucleosomes. Thus, epigenetic changes of the histones would not be (epigenetic) modifications of the DNA molecule. Initially, these various scientific facts seem to argue for also treating epigenetic modifications in DNA legally differently from epigenetic changes in histones. However, with regard to a risk-based consideration for the legal framework, such a different handling does not seem appropriate because the scientific, phenotypic effects of all epigenetic modifications are in fact comparable. Therefore, it is more appropriate if all epigenetic modifications are also treated legally in the same way, regardless of their site of action. However, this uniform handling is not identical with the question whether epigenetic modifications should be legally treated like modifications of the actual DNA, i.e., the question whether epigenetic modification can lead to a GMO. With regard to the question of whether epigenetic modifications, like DNA modifications, lead to a GMO and therefore require regulatory control, it should rather be taken into account that epigenetic modifications are fundamentally different from transgenic modifications.

Firstly, unlike transgenic modifications, epigenetic modifications can never transfer genetic information across the boundaries of organisms or even across the boundaries of biological species. Epigenetic modifications therefore do not allow for genetic combinations that do not or could not occur in nature anyway.

Secondly, epigenetic modifications are also fundamentally different at the level of the individual organism compared to base sequence modifications. Epigenetic modifications cannot activate new properties foreign to the epigenetically altered organism. Only can those properties be switched on or off that are already present in the genetic code of the organism in question. At best, these properties can then be stabilized and emphasized in the context of epigenetic modifications. Moreover, these properties can already occur in the (epigenetically) unmodified organism, i.e., in the wild type, due to the naturally occurring epigenetic fluctuation even without technical influence.

Unlike the transgenesis of DNA, epigenetic modifications do not produce new substances such as toxins or foreign proteins in an organism. Thus, epigenetic changes do not cause those kinds of modifications which, in the case of DNA-based genetic engineering, justify the need for a risk analysis and preventive bans on release by applying the principles of the precautionary principle.

Therefore, for epigenetic modifications (as long as they are not accompanied by modifications of the DNA bases, see below), there is no need for preventive bans on their release *via* a reference to the principles of the precautionary principle as is usual in DNA-based genetic engineering. Rather, in line with the EFSA Statement (2015, cf. Chap 4.2), the scientific, technical and risk-based considerations suggest that epigenetic modifications should legally not be treated like modifications of the DNA. However, it is still questionable whether this technology-based assessment of the legal handling of epigenetically modified organisms is already reflected in the relevant laws today.

### 6.2 Historical arguments

The first approach to the interpretation of the law is the historical interpretation by which the (historical) conception of the legislator is determined. It is, therefore, a question of finding out with what level of knowledge the historic legislator introduced a regulation. In the legislative process for Directive 2001/18/EC, neither epigenetics as such nor epigenetic techniques were addressed, only aspects in relation to the base sequence. This suggests that only the base sequence of DNA is covered by the legal provisions and not epigenetic modifications. The issue with this is that in view of the scientific, technical development, the historical legislator could not consider techniques for epigenetic manipulation.

From a historical perspective, it is, therefore, not possible to answer whether the term “*genetic material*” covers only the base sequence or also epigenetic properties. The same applies to the terms “*altered*” and “*modification*.”

### 6.3 Semantic arguments

Directive 2001/18/EC, as well as the other legislation on genetic engineering, does not contain a definition of the terms “*genetic material*,” “*altered*” or “*modification*.” The first approach to the content of these terms is provided by literal interpretation, which clarifies the semantic meaning of the terms in question.

The term “*genetic material*” typically describes the substrates of genetic information and thus focuses on the hereditary properties of an organism. In higher organisms such as plants, this is at first glance the base sequence of the DNA, but strictly speaking it is the double-stranded DNA in the form in which it contains the genetic information. In contrast, RNA—at least in higher organisms - does not count as genetic material, since RNA (i.e., the information stored in the RNA) is not heritable like DNA.

The genetic, hereditary information is—as shown—not only stored in the sequence of DNA bases, but also in the interaction of base sequence and epigenetic properties. If one takes into account that not only the base sequence is heritable, but also epigenetic properties, this suggests that such epigenetic properties are covered by the term “genetic material.” Moreover, there are no other reasons which argue against including epigenetic properties in the term “genetic material,” because this inclusion does not imply a narrowing of a risk-based evaluation approach based on the precautionary principle. This view also supports the possibility of considering all epigenetic modifications or properties (legally) in a uniform manner.

Since genetic material can be altered with respect to both the base sequence and the epigenetic properties, the term “*altered*” can also refer to altered epigenetic properties. The same applies to the term “*modification*.”

Thus, in an abstract sense, epigenetic modifications may among other things be covered by the term “genetic material.” However, it is still questionable whether the current genetic engineering law also specifically covers the epigenetic techniques in question for generating the epigenetic modifications (without accompanying changes to the DNA base sequence). This can be answered by a systematic interpretation. Even if the meaning is clear from the literal wording, it must be verified whether the result of the literal interpretation is confirmed by the meaning of the words in their context. It is quite possible that the wording only superficially has an unambiguous meaning.

### 6.4 Systematic arguments from directive 2001/18/EC

Art. 2 (2) (a, b), Art. 3 No 1 and the Annexes of Directive 2001/18/EC give quite some hint for the systematic interpretation of the content of the terms “*genetic material*,” “*altered*” and “*modification*.”

First, Annex I with its Part 1 and Part 2 in conjunction with Art. 2(2) (a, b), Art. 3 No 1 lists techniques, which serve as examples of what type of alterations do or do not lead to a GMO in the legal sense. All mentioned techniques deal—at a first glance only—with changes in the DNA base sequence of organisms.

Not just do “*recombinant nucleic acid techniques*” serve as the frontmost example for techniques leading to GMO according to Annex I A Part 1. The exemptions from the GMO definition of Article 2(2) in Annex I A Part 2 also require that they do not involve the use of “*recombinant nucleic acid molecules*,” so do the exceptions to the scope of the Directive according to Article 3 (1) in conjunction with Annex I B.

As shown above, the terms “*genetic material*,” “*altered*,” and “*modification*” may indeed include epigenetic modifications and modifications of the DNA sequence. However, from a systematic point of view, the wording of Annex I with its Part 1 and Part 2 in conjunction with Art. 2 (2) (a, b), Art. 3 No 1 already shows that, contrary to the first literal interpretation, the terms “*genetic material*,”, “*altered*,” and “*modification*” must be restricted to modifications of the DNA base sequence and that these terms are not applicable to epigenetic modifications. This conclusion is appropriate because epigenetic modifications can never be recombinant. However, according to Annex I with its Part 1 and Part 2 in conjunction with Art. 2 (2) (a, b), Art. 3 No 1 DNA recombination is in the light of the precautionary principle a prerequisite that makes regulation by authorities necessary. Therefore, by systematic interpretation and considering the precautionary principle underlying genetic engineering law, Annex I narrows the term “*genetic material*” as well as the terms “*altered*,” and “*modification*” to exclude epigenetic modifications from its scope. Since all epigenetic modifications (unless they are accompanied by modification of the DNA) cannot be transgenic, this restriction excludes all epigenetic modifications (i.e., base-based and histone-based) from the regulation of genetic engineering law. Finally, this uniform exclusion means that all epigenetic modifications without additional DNA modification are handled in a legally uniform manner.

### 6.5 Systematic arguments from comparison with other provisions

A systematic interpretation, which takes into account not only Directive 2001/18/EC but also other legislation regulating genetic engineering, reveals contradictions and questions the legal interpretation expressed in the Commission’s study that epigenetic methods and epigenetically modified organisms are covered by the genetic engineering law.

In fact, the GMO definition in Article 2 (2) of Directive 2001/18/EC is the core of the entire European genetic engineering law. Its interpretation has consequences for other provisions on genetic engineering as well. It is held in legal literature that in case of several possible results of interpretation, the one that fits (best) into the legal context has to be chosen, since it must be assumed that the legislator has created or wanted to create a uniform regulatory system that is free of contradictions. Consequently, in the systematic interpretation of a norm, care must be taken that individual legal propositions which the legislature has placed in a factual context are interpreted in such a way that they are logically compatible with each other (Larenz and Canaris, 1999).

According to Article 5 (3) lit. b), i) and Article 17(3) lit. b), i) Regulation (EC) 1829/2003 the submitted application for authorization of genetically modified food and feed must include the designation of the transformation event(s) as well as methods for their detection and identification.

Since transformation is necessarily associated with the recombination of nucleic acid molecules, there simply are no transformation events for mere epigenetic changes. The legislator obviously interprets transformations as successful DNA-recombinations when the legislator in its own regulation holds that:

“*During the process of the genetic modification of plants and other organisms, marker genes are often used to facilitate the selection and identification of genetically modified cells, containing the gene of interest inserted into the genome of the host organism, among the vast majority of untransformed cells.*”

(European Commission, 2013, recital 18). As has been shown above (cf. chap. 3.3), even if one was to ignore this argument, there currently are no validated methods for detecting organism with epigenetic changes.

Likewise, the scientific information to be provided in applications for placing on the market for genetically modified food and feed also does not fit for mere epigenetic changes. The reason is obvious: the risk level of transgenic GMOs is apparently connected by the legislator to the intended interruption of endogenous nucleotide sequence of an organism (insertion, deletion, mutation by double strand breaks). However, as shown (cf. chap. 6.1), epigenetic modifications cannot be transgenic, thus the risk-based regulation of transgenesis cannot be applied to epigenetic modifications.

This makes sense, since the consequences and impacts of such interruption could be manifold - disruption of endogenous genes, creation of new ORFs (risk of new allergens or toxins), expression of new proteins with unintended effects. Based on these considerations, the legislator created a catalogue of requirements for scientific evaluation of such impacts, which is judged and well thought out. As a whole, however, it only perfectly fits for transgenic organisms as it is based on problem formulation specific for interruption of nucleotide sequence and insertion of recombinant nucleic acid. Problem formulation is a systematic planning step that identifies the key factors to be considered in a particular risk assessment including 1) defining plausible pathways to harm, 2) formulating risk hypotheses about the likelihood and severity of such events and 3) identifying the information useful to test the risk hypotheses. Since the interruption of endogenous nucleotide sequence is not intended by epigenetic modifications, the key factor triggering the risk assessment as defined in Implementing Regulation (EU) 503/2013 fails. The problem formulation for organisms with intended epigenetic modifications would logically result in, if any at all, divergent risk hypotheses and subsequently in divergent data and test requirements. Therefore, applying the GMO legislation as is to epigenetic modifications seems inappropriate and does not contribute to its goals.

So, while Regulation (EC) 1829/2003 generally allows placing on the market of GMO for food and feed purposes following a strict authorization procedure, the interpretation in the study published by the European Commission would factually restrict this possibility, since necessary data for the authorization procedure could not be provided by the applicant.

Finally, in the Commission study it is held that due to the similarity of their respective GMO definitions and the aims of the Directives 2001/18/EC and 2009/41/EC, the CJEU judgment and its interpretation of the GMO directive should also be applicable to Directive 2009/41/EC (“*contained use*”) (European Commission, 2021, p. 21). Consequently, it is being held in the study that organisms with induced epigenetic changes “*are GMOs subject to the* ***provisions of the GMO legislation*** *.*” *[Emphasis added by the author]*, which clearly also refers to Directive 2009/41/EC.

This, however, leads to problems as well: Article 4(2) of Directive 2009/41/EC requires the user to carry out an assessment of the contained uses, i.e., basically any lab-based activity involving GMO as laid down in Article 2(c) of Directive 2009/41/EC, as regards the risks to human health and the environment that those contained uses may pose.

According to Annex III Part A No. 2 of Directive 2009/41/EC, the assessment should be based *inter alia* on the identification of any potentially harmful effects, in particular those associated with the genetic material inserted (originating from the donor organism) and the vector. Obviously, for epigenetic modifications there is neither a donor organism, nor a vector and no inserted genetic material. It, therefore, remains unclear, how a sound risk assessment could be done for epigenetic modifications within the framework of Directive 2009/41/EC.

All of the above shows contradictions arising in the GMO legislation if organisms with mere epigenetic modifications and without additional DNA modification were to be subsumed under the GMO definition. Rather, both the technical and the legal analysis suggest that epigenetic modifications without additional modifications of DNA are not covered by the scope of today’s genetic engineering law. From a legal point of view, therefore, merely epigenetically modified organisms cannot be GMOs.

## 7 Conclusion or a differentiated classification

At first, taking into account the precautionary principle and the associated risk-based evaluation approach, the technical options by which epigenetic modifications (undirected or directed) can be induced in a targeted manner should be classified as follows:

In cases where the epigenetic modification requires an additional change in the base sequence of the DNA, a differentiation must be made between techniques in which the change in the base sequence is permanent and techniques in which the change is only temporarily.

Among the techniques that work without additional modification of the DNA base sequence, a differentiation is necessary between techniques that are comparable to classical, undirected (DNA) mutagenesis by irradiating or chemically treating plants or plant cells and techniques that work by applying nucleic acids (RNA and/or DNA) without integrating these nucleic acids into the DNA of the treated plants (cells). In the latter case, the epigenetic change is only mediated by the applied nucleic acids, without changing the base sequence of the DNA in the treated organism. The applied nucleic acids are degraded after the epigenetic change in the cell by natural processes occurring there (degradation is comparable to RNA-based vaccination in humans).

This classification is justified by the fact that, although the various classes have different risk potentials, the techniques within a class nevertheless have comparable risk potentials. Based on this technical classification, a legal assessment of the individual classifications can then be made:

**Epigenetic modification including permanent change in the DNA base sequence**: For techniques in which epigenetic modification is accompanied by a permanent change in the base sequence of DNA that is not achieved by crossover or natural recombination, the organisms thus generated are regulated GMOs simply by the change to the base sequence. Moreover, no risk-based reasons are apparent why the epigenetic component should override this regulatory assessment.

**Epigenetic modification and temporary change of the DNA base sequence (negative/null segregants)**: For those cases where the epigenetic modification involves only a temporary change in the base sequence of the DNA that is not achieved by crossover or natural recombination, it can be assumed that the organism is a regulated GMO at least during the time of the base sequence change. But what should apply from the moment when the base change is no longer present?

Normally, the progeny of a (legal) GMO is in turn a (legal) GMO, since the progeny carries the genetic modification that made the parent generation a GMO in the legal sense. For the respective progeny, the same safety aspects can be assumed as in relation to the parent generation. As a result, there are no other aspects for the legal assessment. From a legal point of view, therefore, an organism that is derived from a GMO but itself is shown to no longer carry the genetic event in question should not be treated as a GMO. From a scientific point of view, internationally the New Techniques Working Group as well as nationally in Germany the Central Committee on Biological Safety have supported this evaluation in the past (Central Committee on Biological Safety, 2012).

Whether epigenetically modified offspring of null segregants (in relation to changes of the DNA) are GMO from a legal point of view is therefore not only a question on the legal handling of the techniques on epigenetic modifications. Insofar as the progeny of null segregants are legally considered GMO, it is true, as in the case of permanent, lasting changes in DNA, that this legal evaluation is not reversed by an additional epigenetic modification. If, on the other hand, null segregants are not GMOs from a legal point of view, then the epigenetic modification cannot statute a GMO either, because merely epigenetic modifications do not generate GMOs from a legal point of view, according to the view adopted here.

**Epigenetic modification using RNA without changing the DNA base sequence**: In cases where the epigenetic change is mediated by application of RNA without the applied RNA changing the DNA of the target organism (not even temporarily!), a distinction must first be made for the legal assessment between the organism in which the artificially added RNA is present and the organism when the artificially added RNA is no longer present, but the desired epigenetic change has been induced.

Since, at least in higher organisms such as plants, RNA is not part of the genetic material because it is not independently heritable information, the injection of isolated RNA, for example, into plant cells for targeted epigenetic modification and without simultaneous modification of DNA is legally not a GMO neither in terms of a DNA nor in terms of an epigenetic modification. With regard to the DNA, no GMO can exist, since no modification of the DNA is generated. With regard to the epigenetic modification, there is no GMO because merely epigenetic modifications do not generate GMOs from a legal point of view according to the view represented here.

**Epigenetic modification by radiation and/or chemical substances (“*epigenetic mutagenesis*”)**: Theoretically possible, but (so far) without practical significance for undirected epigenetic changes are techniques that, similar to the classical, undirected (DNA) mutagenesis, alter epigenetic properties mediated by chemical and/or radiation-based influence.

Insofar as these techniques become of practical importance, the legal assessment depends on whether the techniques in question only modify the epigenetic properties or whether the epigenetic modification is accompanied by a modification of the DNA.

If the epigenetic changes are associated with modifications of the DNA, the legal assessment is determined by the case law of the CJEU on DNA mutations, whereby from a legal point of view only the modification of the DNA is important, since epigenetic changes alone cannot generate a GMO in the legal sense. Thus, for undirected methods of mutagenesis that were available before the entry into force of Directive 2001/18/EC, the modified organism is a GMO in the legal sense, but is not regulated. Techniques that came onto the market after 2001 would be regulated GMOs. For techniques that only alter epigenetic properties, the CJEU’s case law on mutagenesis and the temporal classification of these techniques is irrelevant, because merely epigenetic alterations *per se* cannot generate a GMO in the legal sense, according to the view expressed here. Therefore, the outcome of the current proceedings before the CJEU on the legal handling of random mutagenesis *in vitro* (Case C-688/21 = Confédération paysanne and Others, *in vitro* random mutagenesis) does not change this assessment. If with the view taken here, merely epigenetically modified organisms cannot be a GMO in the legal sense, then it also does not matter whether the procedure to modify epigenetics is carried out *in vivo* or *in vitro* (randomly or directionally).

## 8 Outlook

As has been shown above, it is legally and scientifically questionable to conclude that the GMO definition in Article 2 (2) of Directive 2001/18/EC must be interpreted as to cover organisms with mere epigenetic modifications—even in light of the CJEU judgment. The precautionary principle and the Directive’s objectives do not necessarily demand so.

The legal classification expressed in the EU Commission study, according to which mere epigenetic modifications generally lead to GMO seems too sweeping considering how differently epigenetic techniques modify organisms in comparison to transgenesis and site-directed-nucleases. It also is not accompanied by a thorough reasoning.

The legal question on the applicability of the GMO legislation to organisms with mere epigenetic modifications should, therefore, in the light of technical progress be discussed as a multilateral effort on a European level to provide legal certainty.

Irrespective of the question of the classification of epigenetically modified organisms under genetic engineering law, it should also be clarified whether, especially in the case that epigenetically modified organisms are not GMOs in the legal sense, existing food law such as Regulation (EC) 178/2002 on General Food Law or Regulation (EU) 2015/2283 on Novel Foods adequately regulate epigenetically modified organisms with regard to their use as food and feed.
